# Development and Evaluation of a Digital Intervention for Fulfilling the Needs of Older Migrant Patients With Cancer: User-Centered Design Approach

**DOI:** 10.2196/21238

**Published:** 2020-10-26

**Authors:** Hande Sungur, Nida Gizem Yılmaz, Brittany Ming Chu Chan, Maria E T C van den Muijsenbergh, Julia C M van Weert, Barbara C Schouten

**Affiliations:** 1 Department of Communication Science Amsterdam School of Communication Research/ASCoR University of Amsterdam Amsterdam Netherlands; 2 Department of Public and Occupational Health Amsterdam UMC Vrije Universiteit Amsterdam Amsterdam Netherlands; 3 Department of Primary and Community Care Radboud University Medical Centre Nijmegen Netherlands

**Keywords:** cancer, patient participation, health services needs and demand, eHealth, migrants, physician-patient relations, culture, mobile phone

## Abstract

**Background:**

Older migrant patients with cancer face many language- and culture-related barriers to patient participation during medical consultations. To bridge these barriers, an eHealth tool called *Health Communicator* was developed in the Netherlands. Essentially used as a digital translator that can collect medical history information from patients, the Health Communicator did not include an oncological module so far, despite the fact that the prevalence of Dutch migrant patients with cancer is rising.

**Objective:**

This study aims to systematically develop, implement, and conduct a pilot evaluation of an oncological module that can be integrated into the Health Communicator to stimulate patient participation among older Turkish-Dutch and Moroccan-Dutch patients with cancer.

**Methods:**

The Spiral Technology Action Research model, which incorporates 5 cycles that engage key stakeholders in intervention development, was used as a framework. The *listen* phase consisted of a needs assessment. The *plan* phase consisted of developing the content of the oncological module, namely the question prompt lists (QPLs) and scripts for patient education videos. On the basis of pretests in the *do* phase, 6 audiovisual QPLs on *patient rights*, *treatment*, *psychosocial support*, *lifestyle and access to health care services*, *patient preferences*, and *clinical trials* were created. Additionally, 5 patient education videos were created about *patient rights*, *psychosocial support*, *clinical trials*, and *patient-professional communication*. In the *study* phase, the oncological module was pilot-tested among 27 older Turkish-Dutch and Moroccan-Dutch patients with cancer during their consultations. In the *act* phase, the oncological model was disseminated to practice.

**Results:**

The *patient rights* QPL was chosen most often during the pilot testing in the *study* phase. Patients and health care professionals perceived the QPLs as easy to understand and useful. There was a negative correlation between the tool’s ease of use and patient age. Patients reported that using the module impacted the consultations positively and thought they were more active compared with previous consultations. Health care professionals also found patients to be more active than usual. Health care professionals asked significantly more questions than patients during consultations. Patients requested to see the *patients’ rights* video most often. Patients rated the videos as easy to understand, useful, and informative. Most of the patients wanted to use the tool in the future.

**Conclusions:**

Older migrant patients with cancer, survivors, and health care professionals found the oncological module to be a useful tool and have shown intentions to incorporate it into future consultation sessions. Both QPLs and videos were evaluated positively, the latter indicating that the use of narratives to inform older, low-literate migrant patients with cancer about health-related topics in their mother tongue is a viable approach to increase the effectiveness of health care communication with this target group.

## Introduction

### Background

Migrant patients across the world are facing numerous adversities within the health care system. For instance, health care professionals communicate less adequately with migrant patients compared with nonmigrant patients [1], and their affective (ie, relating to psychosocial issues such as psychological support) and instrumental needs (ie, relating to more medically related issues such as being informed about treatments) are often not fully met [2]. Health care professionals particularly tend to overlook the importance of ensuring that migrant patients in language-discordant consultations comprehend all the information presented. This can induce dissatisfaction among migrant patients and their family members, ineffective consultation sessions, and ultimately worse patient health outcomes compared with language-concordant consultations [3]. In addition to language barriers, unresolved cultural barriers, such as culturally shaped beliefs about health and illness and communication styles, might also provoke migrant patients’ perceptions of lack of health care professionals’ respect, inability to participate in the decision-making process, and even perceived inequalities and discrimination in care quality [4-6].

The number of Dutch migrant patients with cancer is expected to triple within the next 20 years, mainly because of the aging of the first generation Turkish-Dutch and Moroccan-Dutch population [7]. However, cancer rates are still low among Dutch migrant patients with cancer; nevertheless, it becomes increasingly important to address language and cultural barriers to adequate health care professional-patient communication and enhance older migrant patients’ participation during medical consultations in oncological care. Patient participation refers to “the extent to which patients produce verbal responses that have the potential to significantly influence the content and structure of the interaction as well as the healthcare professional’s beliefs and behaviours” [8]. In particular, when it comes to the first generation Turkish-Dutch and Moroccan-Dutch patients with cancer, who often prefer to take along relatives to their consultation to bridge the language barrier instead of professional interpreters, there is a risk of relatives inhibiting patient participation during medical consultations because of culturally shaped beliefs about communication and treatment [9], resulting in providers not getting the patient information they need to provide good quality health care. Hence, interventions that address these barriers are urgently needed.

In general, patient-targeted intervention strategies that have been found to enhance patient participation significantly include coaching, providing educational materials, and having patients offer their opinions to their health care professionals [10,11]. An example of a successful intervention that employed the abovementioned strategies comes from Epstein et al [12]. The results of this intervention, which was neither targeted at migrant patients nor meant to address language barriers, showed that more patient-centered communication took place during consultations with patients who received question prompt lists (QPLs) and individualized communication coaching, which assisted patients in identifying issues that needed to be addressed during consultation sessions, than during consultations with patients who did not receive these communication strategies. In addition, previous research indicated that a written QPL is beneficial to the communication and psychological and cognitive outcomes of patients with cancer [13].

However, Fukui et al [14] concluded that interventions need to be remodeled to align with the cultural characteristics of their participants to yield satisfactory results. In an intervention tailored to Americans, patients with cancer were provided with statistical information such as recurrence and survival rates. However, information related to the truth of illnesses in Japan was frowned upon among the Japanese participants. In the modified version, patients with cancer were given the liberty to ask for medical information in their own terms. Upon modification, more than 80% of Japanese patients with cancer expressed high satisfaction with the intervention, and no participants dropped out of the intervention compared with a 30% dropout rate in the American version of the intervention. Hence, the results of this study show that interventions must be culturally tailored to be effective.

### This Study

Given the lack of interventions tailored specifically to older Turkish-Dutch and Moroccan-Dutch migrant patients with cancer to combat the barriers they experience in communicating with their health care professionals [15,16], this study set out to systematically develop and implement an intervention to improve their participation and satisfaction with care. As the Turkish and Moroccan community has been growing as a result of migration, and recent figures have shown that they not only make up approximately 5% of the Dutch population but also account for being two of the fastest-growing populations in the last 5 years [17,18], we focused on older Turkish-Dutch and Moroccan-Dutch patients with cancer (>55 years). We developed an intervention, more specifically an oncological module, to be implemented in an existing Dutch eHealth tool called *Health Communicator.* The Health Communicator is a web-based digital tool that aims to resolve language barriers between non-Western patients with low Dutch language proficiency and their health care professionals. The Health Communicator is used to collect patient medical anamnestic data via digital questionnaires and to provide educational videos for patients in multiple languages about their illness. Although the Health Communicator includes medical history questionnaires and educational videos for various medical topics (eg, diabetes, pregnancy), it lacked a module specifically aimed at oncology patients, especially one systematically developed for older Turkish-Dutch and Moroccan-Dutch migrant patients with cancer (Multimedia Appendix 1).

Our goal of implementing this new oncological module is further supported by evidence pointing toward the fact that older adults and Turkish-Dutch patients are more inclined to use media than interpersonal sources to gather health information, ranging from the television to the internet [19]. In a recent study, health care professionals have expressed positive attitudes and intentions to use the existing Health Communicator tool to increase patient participation (Yilmaz et al, unpublished data, 2020). Furthermore, older adults display more competence in recalling health information in an audiovisual format than a text-only format [20]. Hence, the overall aim of this study was to systematically develop, implement, and conduct a pilot evaluation of a web-based oncological module that can be integrated with the Health Communicator to stimulate patient participation among older Turkish-Dutch and Moroccan-Dutch patients with cancer.

## Methods

The Spiral Technology Action Research (STAR) [21] model was used as the guiding framework for developing the oncological module. This model encourages several evaluation cycles among stakeholders, enabling continuous improvement of the intervention until it is finalized. To establish a high degree of relevance for target users, the model incorporates 5 guiding cycles that engage relevant community members in developing the intervention. These 5 cycles, *listen*, *plan*, *do*, *study*, and *act*, continuously weave technological and community development together [21]. In the following sections*,* we describe the steps taken in each phase with a special emphasis on the study phase and its results. Table 1 provides an overview of the aims and actions during each phase.

**Table 1:** Overview of the phases, their aims, actions, and publications.

Phase	Aim	Actions	Publication
Listen	Target group analysis	Conduct a literature review to identify (older) ethnic minority cancer patients’ information and participation preferences and needs Conduct a qualitative study to identify unfulfilled instrumental and affective needs of older Turkish-Dutch and Moroccan-Dutch patients with cancer, and the barriers perceived by health care professionals (ie, general practitioners and oncology nurses) to fulfil patients’ needs	Review [22] Submitted; paper under review (Yilmaz et al, unpublished data, 2020)
Plan	Methodology development	Develop question prompt lists and pilot testing them among patients Developing narrative patient education videos and pilot testing them among both patients and health care professionals (ie, general practitioners and oncology nurses)	Results shortly described in the Methods section of this paper
Do	Development of the oncological module prototype	Finalize the 6 question prompt lists and 5 narrative patient education videos by revising them based on feedback from patients and health care professionals (ie, general practitioners and oncology nurses) from the *plan* phase	No publication
Study	Pilot evaluation of the oncological module	Conduct a qualitative study to pilot-evaluate the oncological module prototype among patients and health care professionals (ie, general practitioners and oncology nurses)	Findings described in the Results section of this paper
Act	Creating a dissemination plan	Create and disseminate a stand-alone module: *the Conversation Starter*	No publication

### Listen: Target Group Analysis

To identify existing findings on the needs of older migrant patients with cancer, a systematic literature review was conducted. The results of the literature review revealed that most non-Western ethnic minority patients with cancer and survivors have a high preference and need for information and shared or active participation. However, no information was available regarding the preferences and needs of our target population, Moroccan-Dutch and Turkish-Dutch older patients with cancer [22].

Given the limited findings in the literature regarding the needs of older Turkish-Dutch and Moroccan-Dutch patients with cancer, further in-depth interviews were conducted to determine the topics that need to be addressed within the oncological module. A total of 19 interviews were carried out with Turkish-Dutch and Moroccan-Dutch patients with cancer and cancer survivors. Interviews were conducted by bilingual interviewers in patients’ first language (ie, Turkish, Moroccan Arabic, or Berber dialects). Participants were recruited by reaching out to prominent figures within Turkish and Moroccan communities that work in the health sector and via snowball sampling. The results revealed unmet instrumental needs concerning the treatment of cancer and the health care system and unmet affective needs concerning psychosocial support and affective doctor-patient communication. Acceptance of the Health Communicator*,* which was studied based on concepts of the Technology Acceptance Model (TAM), revealed that patients thought that the Health Communicator would be useful to fulfill their unfulfilled needs, but ultimately did not intend to use the tool (Yilmaz et al, unpublished data, 2020).

Following patient interviews, 2 focus groups with general practitioners (GPs) and oncology nurses were conducted. Health care professionals were asked to reflect on the findings of the patient interviews, share their own experiences, and indicate the type of information they would like to receive from their patients to better fulfill their unmet needs. Although health care professionals acknowledged the most unmet instrumental and affective needs of patients that emerged from the interviews, they appeared not to be aware of patients’ need for psychosocial support or misunderstandings surrounding clinical trial requests. Health care professionals also needed more information about patients’ instrumental needs and the role of family members in the treatment process (Yilmaz et al, unpublished data, 2020). Overall, the patient interviews and focus groups with GPs and oncology nurses provided valuable insights into the content creation phases.

### Plan: Methodology Development

On the basis of the results of the target group analysis, 2 types of content, namely QPLs and narrative patient education videos, were created for the oncological module. QPLs are essentially structured question lists that aim to make it easier for patients to ask questions to their providers during consultations [13]. Typically, patients can look at these premade lists before the consultations and select the questions they would like to ask their health care providers during consultations. Selecting questions from a premade list is easier than formulating the questions on their own for the patients. In addition, selecting them before consultations decreases the risk of forgetting to ask them during consultations because of time pressure or other distractions. Narrative patient education videos that depict patient stories are increasingly used in health communication as they are shown to enhance learning, recall of information, and intentions to stimulate healthy behavior and attitudes [23]. They are especially suitable for relaying cancer-related information as they can transport the patient into the story and make it easier for them to identify with the positive role models in the videos [24]. In the methodological development phase, several pilot tests with patients and professionals were conducted for the QPLs and videos before moving on to the third phase where the prototype was built.

#### Developing QPLs

On the basis of the emergent instrumental and affective needs of patients, 5 QPLs were developed. The content of the QPLs was not disease-specific (ie, not about breast cancer or other cancer types) but rather included topics relevant to general oncological care that addresses the issues that older migrant patients with cancer face during their illness, namely (1) patient rights, (2) lifestyle and access to health care services, (3) treatment, (4) psychosocial support, and (5) clinical trials. Each QPL consisted of 4 or 5 simple questions aimed at stimulating patients to ask more questions to their health care professionals and have a more active role during consultations (eg, “Can I discuss my problems in my mother tongue with someone sharing my culture?” and “Can I ask for a second opinion?”). In addition, to address health care professionals’ instrumental needs, a sixth QPL was developed, which enabled them to learn more about patients’ instrumental and decision-making preferences and health behaviors (eg, “Are you using any medication bought from another country?” and “Who do you want, next to your doctor, to help you make healthcare decisions for you?”). Additional illustrations representing each question were developed to assist patients with low literacy.

All QPLs and accompanying illustrations were pilot-tested during in-depth interviews conducted with 11 older migrant patients with cancer and survivors (8 Moroccan and 3 Turkish; mean age 61.50, SD 9.36 years). Participants were recruited by reaching out to prominent figures within Turkish and Moroccan communities that work in the health sector and via snowball sampling.

Interviews were conducted by bilingual interviewers in the patients’ first language (ie, Turkish, Moroccan Arabic, or Berber dialects). Patients evaluated each question and illustration for ease of understanding of the content and language (eg, “Do you understand this question?” and “Do you think this picture is a clear illustration of the question?”). Results showed that patients found the QPL questions easy to understand, whereas some illustrations were found to be too abstract. Patients had specific recommendations for word choices and requested increasing concreteness and familiarity of illustrations, such as adding headscarves to some of the female figures and removing abstract symbols. On the basis of their recommendations, revisions were made and incorporated in the *do* phase of our intervention development.

#### Developing Videos

We created 5 scripts for each video. Similar to QPLs, the content of the videos addressed general oncological issues that older migrant patients with cancer typically face during their illness, namely (1) patient rights and access to health care services, (2) doctor-patient communication, (3a and 3b) psychosocial support, and (4) clinical trials. We created 2 separate scripts featuring a male and female patient as the main characters for the psychosocial support video. This was done to enhance identification with characters for both male and female patients. Each script was related to the experiences of an older migrant patient who survived cancer from the patients’ point of view. The scripts incorporated actual experiences and specific language used by patients during interviews in the target group analysis phase as much as possible. All scripts were prepared in Dutch for health care providers and in Turkish, Moroccan Arabic, and Berber dialects for patients.

The scripts were pilot-tested with 8 older migrant patients with cancer (Turkish, n=3; Moroccan, n=5; mean age 63.75, SD 6.39 years) during individual in-depth interviews. Participants were recruited by reaching out to prominent figures within Turkish and Moroccan communities that work in the health sector and via snowball sampling. Interviews were conducted by bilingual interviewers in patients’ first language (ie, Turkish, Moroccan Arabic, or Berber dialects). Patients evaluated each script on ease of understanding (“Can you understand everything said in the video/happening in the video easily?”), familiarity (“Does this story sound familiar to you?”*),* usefulness (“Do you find the information provided in the story useful?”), believability (“Do you find the information believable?”), emotions induced (“Does this story induce any emotion for you and if yes what type of emotions?”), and level of identification with the main characters (“Can you put yourself in the shoes of the character in the story?”). Patients found the scripts easy to understand and reported very high familiarity, believability, and identification with the characters. They stated that they could see themselves in these stories and understand the emotions shared by the characters, and they also found them to be very useful for other patients.

We also tested the scripts in 2 separate focus group meetings with GPs (n=6 [2 women and 4 men]; mean age 45.17, SD 11.89 years) and oncology nurses (n=5, all women; mean age 49.60, SD 12.16 years). The health care professionals evaluated the scripts on accuracy (“Is all the information provided in the script correct?”), the usefulness of the provided information (“How useful do you find the information in the script?”), and their willingness to share videos once they are available (“Would you share these videos with your patients in the future?”). The results showed that health care professionals found the scripts to be accurate and useful, and they expressed their intentions to use them in the future.

### Do: Developing the Oncological Module Prototype

On the basis of the results of the pilot tests with patients and health care professionals, we created a prototype for the oncological module. The prototype included 6 QPLs tested in the previous phase. We used voice actors and added audio support to the QPLs in Turkish, Moroccan Arabic, and Berber (Tarafit dialect), enabling (illiterate) patients to listen to the QPLs in their mother tongue.

Similarly, feedback on the scripts was incorporated, and 5 short videos (1.5-3.5 min long) featuring Turkish-Dutch and Moroccan-Dutch actors were filmed. Once again, voice actors narrated these stories in Turkish, Moroccan Arabic, and Berber (Tarafit). Scripts were largely based on patients’ answers during the interviews in the target group analysis. The first video addressed patients’ instrumental needs about patient rights (eg, right to informed consent) and access to health care services (eg, dietitian, home care, psychological support). The second video included suggestions to improve GP-patient communication by encouraging patients to prepare before consultations, ask more questions, make use of interpreters, and inform the doctor about their affective needs. The third and fourth videos aimed to provide psychosocial support to patients. The videos acknowledged the negative emotions experienced by patients with cancer and tried to counter them by giving a positive but also realistic message of hope. Topics about self-care, such as the importance of good diet, social contacts, psychological and spiritual support, were incorporated into these videos. The final video aimed to clarify the misunderstandings surrounding clinical trial requests; the video emphasized that a request to join a clinical trial does not mean that the patient has no hope of treatment and that patients can take time to decide on joining these trials, can refuse to join without risking their relationship with their doctors, and can quit if they have accepted to join previously. Furthermore, information regarding the general aim of these trials was provided.

### Study: Pilot Evaluation of the Oncological Module

#### Sample

The oncological module was pilot-evaluated in practice among 27 Turkish-Dutch and Moroccan-Dutch older patients with cancer aged 50 years and older and cancer survivors (Turkish: n=15, 9 women, mean age 63.47, SD 2.59 years; Moroccan: n=12, mean age 63.33 years, SD 2.70 years) and their health care professionals (GPs and oncology nurses: n=12, mean age 53.50 years, SD 13.34 years; see Tables 2 and 3 for sample characteristics). Dutch language proficiency was self-reported by patients on a 4-point scale (1=poor, 2=mediocre, 3=reasonable, and 4=good). Patients were recruited by first targeting their health care professionals; on the basis of snowball sampling, with the help of health care professionals who participated in the earlier phases of the study, we reached other health care professionals, who then invited their patients to participate in the study.

**Table 2:** Background characteristics of patients.

Demographics		Turkish (n=15)	Moroccan (n=12)	Total (n=27)
Age (years), mean (SD)		63.47 (2.59)	63.33 (2.70)	63.41 (9.55)
Years residing in the Netherlands, mean (SD)		39.40 (3.21)	34.09 (3.54)	37.15 (12.19)
Dutch language proficiency, mean (SD)		1.89 (0.30)	2.21 (0.29)	2.02 (1.08)
**Sex, n (%)**				
	Female	9 (60)	9 (75)	18 (67)
	Male	6 (40)	3 (25)	9 (33)
**Education level, n (%)**				
	No schooling	4 (26)	6 (50)	10 (36)
	Primary school in Turkey or Morocco	7 (47)	5 (40)	12 (44)
	Secondary school in Turkey or Morocco	1 (7)	—^a^	1 (4)
	Primary school in the Netherlands	—	1 (10)	1 (4)
	Secondary school in the Netherlands	2 (13)	—	2 (8)
	Higher education in the Netherlands	1 (7)	—	1 (4)

^a^No participants belonging to the category.

**Table 3:** Background characteristics of health care professionals.

Demographic		General practitioners (n=10)	Oncology nurses (n=2)	Total (n=12)
Age (years), mean (SD)		57.50 (10.50)	33.50 (2.12)	53.50 (13.34)
Work experience in years, mean (SD)		23.44 (10.37)	6.00 (2.82)	20.27 (11.67)
**Sex, n (%)**				
	Female	5 (50)	2 (100)	7 (58)
	Male	5 (50)	—^a^	5 (42)
**Number of older patients with cancer aged ≥50 years with a Turkish or Moroccan background treated in the previous 2 years**				
	1-2	1 (10)	—	1 (8)
	2-4	4 (40)	—	4 (30)
	5-10	5 (50)	1 (50)	6 (50)
	≥10	—	1 (50)	1 (8)

^a^No participants belonging to the category.

#### Procedure

**Figure 1:**
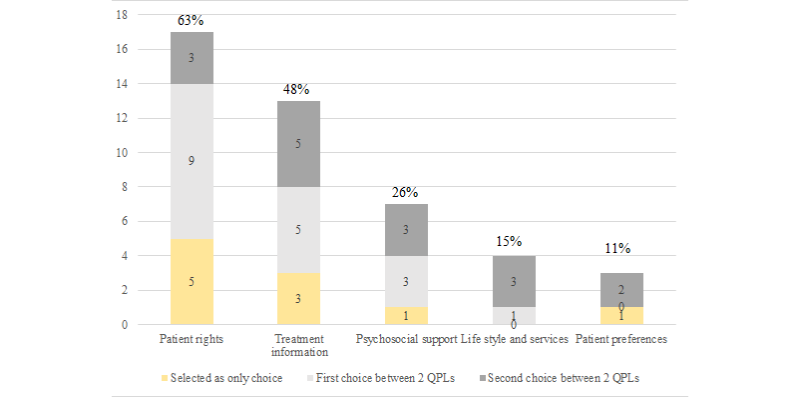
Breakdown of question prompt lists selected by the patients in the evaluation study. Percentages reflect the percentage of participants that selected that question prompt list. Among the 27 participants, 10 selected 1 and 17 selected 2 question prompt lists. QPLs: question prompt lists.

Before the beginning of the pilot testing with patients, all participating health care professionals received an hour-long individual training on how to use the oncological module. Patients evaluated the QPL content and use before their consultations in GP practices or hospitals by means of surveys that were verbally administered to patients in their mother tongue by bilingual trained interviewers. Interviewers presented patients with QPL themes in the oncological module and asked them to choose one or more QPLs that they would like to fill out. After choosing a theme (eg, patient rights), patients saw all available questions within that QPL (eg, “Can I record consultations?”) and selected the ones they wanted to discuss with their professionals during the consultation (Figure 1). Before the consultation started, patients evaluated their experience with the QPLs that they chose. Health care professionals rated the usefulness and ease of use of QPLs at the end of the pilot study.

Following the QPL selection, patients and professionals consulted the same GP practice or hospital; 18 patients gave consent to have their consultations audio-taped. Patients who did not consent felt that the topics were too private and did not want anyone else to hear them, although their anonymity and confidentiality were guaranteed. After the consultations, patients completed a survey again, measuring their evaluation of the consultation.

**Figure 2:**
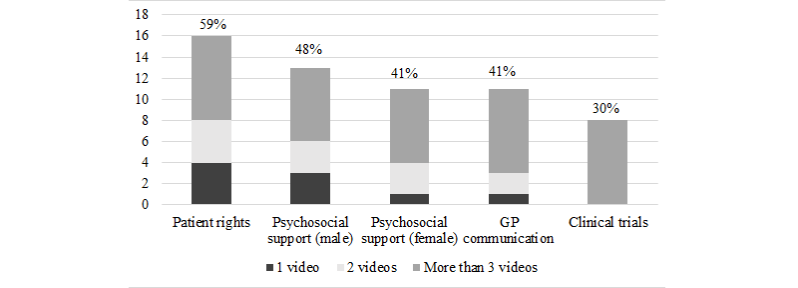
Videos selected by the patients. Percentages reflect the percentage of participants that selected that video. 33% of patients selected 1 video, 22% selected 2 videos, 4% selected 3 videos, 19% selected 4 videos, and 11% selected 5 videos, amounting to an average of 2.27 (SD 1.59) videos requested per patient. GP: general practitioner.

After the consultations, the topics of all available narrative videos were briefly described to patients. Patients selected the videos they wanted to watch (Figure 2). Most of the patients preferred to receive videos on their smartphones. When this was not possible, patients requested that the videos were sent to patients’ family members’ phones. After patients watched the videos, phone interviews were conducted within a week, on average, to assess how they perceived the videos. During the same phone interviews, patients were asked to rate the overall usefulness of the oncological module for improving their communication with their health care providers (ie, “Overall, this tool is useful to improve my communication with my healthcare provider”). Health care professionals also responded to a similar question during the survey, which they filled at the end of their participation (ie, “The oncological module is useful in bridging communication barriers between migrant patients and their providers”).

#### Measures

All survey items were measured using 5-point Likert scales (eg, 1=completely disagree, 5=completely agree; 1=very difficult, 5=very easy; and 1=very unsatisfied, 5=very satisfied).

##### Patients’ Evaluation of QPLs

After making their selection, patients assessed the ease of using the tool (both with and without assistance), ease of understanding QPL content and accompanying graphic images, and finally how convenient it was to choose QPLs before a consultation.

##### Patients’ Evaluations After Consultation

Patients’ general satisfaction with the consultation was measured with 1 item (ie, “Overall, how satisfied are you with your consultation?”). Patients’ perceptions of the informativeness and supportiveness of the health care professional were measured by 3 and 4 items, respectively, based on Street et al [25] (informativeness Cronbach α=.84; supportiveness Cronbach α=.95). Patients’ attitudes toward the oncological module were measured with 2 items (using this tool “…made communicating with my doctor or nurse easier than usual” and “…had a positive influence on my consultation with my doctor or nurse”; Cronbach α=.87) and intention to use it again was measured with 1 item (“I would like to use this tool again in the future”) [25].

Patients’ perceived participation during the consultation was measured using 5 items from Street et al [25] adaptation of the Lerman et al [26] perceived involvement in care scale (PICS; eg, “I asked my healthcare professional to explain the discussed topics in detail”; Cronbach α=.82). An additional question was used to measure the level of participation in comparison with previous consultations (ie, “I felt I could participate more than usual in this consultation”).

##### Patient Participation

Patient participation was assessed with a self-developed codebook containing 2 measures: (1) relative talk for each person (ie, patient, health care professional, interpreter or companion) and (2) number and type of questions asked by patients, professionals, and interpreters or companion. In 11 of 18 consultations, an informal interpreter and/or companion was present; 8 consultations were held by nurses, 10 by GPs. Questions were divided according to the QPLs, except for clinical trials because this QPL could not be chosen because it was deemed irrelevant for GPs (ie, patient rights, treatment-related information, psychosocial support, lifestyle and access to health care services, patient information). In addition, a *miscellaneous* category was added to questions unrelated to these categories (eg, social questions, checking understanding, etc). The number of questions was counted per speech turn. If more than one question per turn was asked to address the same topic, this was counted as 1 question. Rhetorical questions were not addressed. Interpreters or companions’ questions that were a translation of a patient’s or professional’s questions were not counted.

##### Patients’ Evaluation of the Narrative Videos

During the phone interviews, patients rated how easy it was for them to access (“How easy was it for you to access the videos”) and understand the videos (“The video was easy to understand”) and the usefulness (“The video was useful to improve my understanding of the topic about…”) and informativeness (“The video was very informative concerning the topic about…”) of each video that they had seen. The level of identification with the main characters in the narrative videos was measured with 4 items (Cronbach α=.81); 2 items were based on Murphy et al [24] and measured similarity and liking of the main characters. The other 2 items were based on the Cohen [27] identification scale and measured empathy for emotions experienced by the main characters. Finally, the patient’s intention to watch similar videos in the future was measured (ie, “In the future, would you like to watch videos that are similar to the ones that you watched?”) and whether they mentioned the videos to others (eg, friends, family members).

##### Health Care Professionals’ Evaluation

After each consultation, health care professionals evaluated the participation of the patients during the consultation. To measure this, the same questions that patients responded to when reporting their participation were used. Specifically, these were the 5 items from the PICS (eg, “The patient asked me to explain the discussed topics in detail”; Cronbach α=.74) and the additional question about comparison of the participation level (ie, “The patient participated more than usual in this consultation”). During the final survey study, health care professionals also evaluated the QPLs and narrative videos on perceived usefulness, ease of sharing with patients, and their intentions to use in the future.

### Analysis

Correlations between variables were calculated by running bivariate correlations using Pearson *r* as the correlation coefficient. The relative talk was measured by counting all words per person and calculating the word ratio. Question-asking was analyzed by means of descriptive analyses (mean and SD). Differences in the amount of question-asking were assessed using paired-samples *t* tests. The relationship between the first QPL topic choice by the patients and the number of questions asked by patients and health care professionals about that topic during consultations were assessed by simple linear regression analyses. Interrater reliability was calculated for 5 of 18 transcripts (27.7%; two-way mixed-effects model) and was 0.99 for professionals’ questions, 1.0 for patients’ questions, and 0.99 for interpreter or companions’ questions.

### Act

After the *study* phase was completed, a dissemination plan was devised in collaboration with the Dutch expertise center on migrant health care, Pharos, to distribute the oncological module to target audiences. First, to clearly communicate the goal of the oncological module, it was named the *Conversation Starter*. Next, the key organizations and actors that could help us reach health care professionals and older migrant patients with cancer were identified. Different newsletters highlighting the relevant parts of the Conversation Starter were prepared for these different audiences. Finally, a web version of the Conversation Starter that can be freely accessed without the Health Communicator application was created [28].

## Results

### Evaluation of QPL Content and Use

#### Patients

Overall, patients perceived the questions in the QPLs as easy to understand (mean 4.30, SD 0.65) and useful (mean 3.96, SD 0.72). Graphic images accompanying the QPLs were perceived as easy to understand (mean 3.75, SD 1.14). Patients found the usefulness of the images in aiding the understanding of the questions as neutral (mean 3.23, SD 1.27). Overall, participants found it relatively easy to use the QPL function in the oncological module (mean 3.54, SD 1.07). They had low confidence, although still neutral, about their ability to use the tool easily without any assistance (mean 3.19, SD 1.44). There was a strong negative correlation between the ease of using the tool and patient age. It was harder for older patients to use the QPLs (*r*=−0.48; *P*=.01), and they were also less confident in their ability to use the tool without any assistance (*r*=−0.67; *P*<.001). Approximately 70% of the patients reported that it was convenient for them to select the QPLs before their consultations (mean 3.85, SD 1.35). Similarly, younger age was related to reporting more convenience in using the QPL function before consultations (*r*=−0.56; *P*=.003).

#### Professionals

Health care professionals rated the QPLs as rather useful (mean 3.67, SD 1.07) and overall somewhat easy to use (mean 3.44, SD 0.96).

### Patient Participation

The mean consultation length was 14.09 min (SD 7.60; range 3.37-35.06 min). Professionals spoke, on average, most words (48.15%; range 166-2481), followed by patients (33.5%; range 130-2544), and interpreters or companions (18.35%; range 38-1672). The mean number of questions asked per consultation was 24.55 (SD 17.21; range 11-89). Professionals asked significantly more questions (mean 15.72, SD 9.36; range 3-43) than both patients (mean 4.56, SD 5.45; range 0-23; t_17_=6.61; *P*<.001) and interpreters or companions (mean 4.28, SD 6.44; range 0-23; t_17_=4.81; *P*<.001). The most asked questions were about treatment-related information (mean 9.89, SD 15.87), followed by questions on miscellaneous topics (mean 9.28, SD 4.34), patient rights (mean 2.17, SD 2.04), psychosocial support (mean 1.67, SD 2.97), and lifestyle and access to health care services (mean 1.50, SD 2.75). The least asked questions were about patient information (mean 0.06, SD 0.24).

### Relationship Between QPL Choice and Participation During Consultations

#### Patients

Overall, no significant relationship was found between the first QPL selection and the number of questions asked by patients during the consultation about that topic (patient rights: *R*^2^=0.17, b*=0.12; t_16_=0.68, *P*=.51; 95% CI −0.267 to 517; treatment: *R*^2^=0.05, b*=−0.01; t_16_=−0.20, *P*=.84; 95% CI −0.058 to 0.048; and psychosocial support: *R*^2^=0.15, b*=−0.11; t_16_=−0.60, *P*=.56; 95% CI −0.502 to 0.279). As there were not enough patients who selected the QPLs about lifestyle and access to health care services and patient preferences as their first choice, it was not possible to run the analyses to test these relationships.

#### Professionals

Similarly, we tested the relationship between patients’ first QPL choice and the number of questions posed by health care professionals about that topic. The number of questions asked by the health care professionals in a given topic was marginally significantly predicted by the first QPL topic selected by the patients for patient rights (*R*^2^=0.42, b*=0.14; *t_16_*=1.85, *P*=.08; 95% CI −0.021 to 309) but not for treatment (*R*^2^=0.09, b*=−0.01; *t_16_*=−0.37, *P*=.72; 95% CI −0.040 to 0.028) and psychosocial support (*R*^2^=0.15, b*=0.02; *t_16_*=0.61, *P*=.55; 95% CI −0.060 to 0.108).

### Evaluation of Consultations

#### Patients

Overall, patients reported being highly satisfied with their consultations (mean 4.31, SD 0.55). They perceived their providers as informative (mean 4.17, SD 0.57) and supportive (mean 4.34, SD 0.59). They also reported that using the module positively impacted consultations (mean 3.90, SD 0.55). Patients perceived themselves to be rather active during the consultations (mean 3.75, SD 0.84) and thought they were somewhat more active in comparison with previous consultations (mean 3.75, SD 0.79). Most (70%) of the participants reported that they would like to use the tool in the future (mean 3.85, SD 1.25). The age of the participants had a strong negative correlation with their wish to use the tool in the future (*r*=0.45; *P*=.02).

#### Professionals

Professionals’ evaluation of the patients’ participation revealed similar, although slightly lower scores. Professionals found patients to be slightly more active in asking questions and expressing themselves (mean 3.42, SD 0.70) and somewhat more active than their usual levels of participation (mean 3.61, SD 1.06). Both patients’ and providers’ evaluations were positively correlated for each of these measures (*r*=0.58, *P*=.002 and *r*=0.64, *P*<.001, respectively).

### Evaluation of Narrative Videos

#### Patients

Patients rated the videos as easy to understand (mean 4.30, SD 0.47), useful (mean 4.00, SD 0.62)*,* and informative (mean 4.20, SD 0.57). Identification with the characters in the videos was high (mean 3.93, SD 0.54). Patients were somewhat motivated to watch similar videos in the future (mean 3.55, SD 0.85). Around 30% of the patients talked about videos with others (ie, told their friends and family members that they watched them).

#### Professionals

Professionals rated the videos as useful (mean 3.92, SD 1.08). They were neutral about the ease of sharing the videos with their patients (mean 3.25, SD 1.06) and showed some intentions to share them (mean 3.42, SD 1.08).

### Overall Evaluation of the Oncological Module

Overall, patients found the tool to be useful in improving their communication with their health care professionals (mean 4.27, SD 0.88). Health care professionals rated the usefulness of the oncological module less favorable than the patients (mean 3.42, SD 1.16).

## Discussion

### Principal Findings

This paper elaborates on the development of an oncological module aimed at increasing patient participation between older migrant patients with cancer and their health care professionals. Using the STAR model as our framework, the module was developed with continuous input from relevant community members, namely older migrant patients with cancer, oncology nurses, GPs, and researchers. This multifaceted contribution allowed us to develop a multilingual intervention that received positive evaluations from both migrant patients with cancer and professionals. Patients most often chose the QPL for patient rights. Both patients and health care professionals perceived the QPLs as somewhat easy to understand and useful. The correlation between the ease of using the tool and patient age was negative. Health care professionals asked significantly more questions than patients, whereas patients reported that using the module impacted the consultations positively and thought they were more active compared with previous consultations. Health care professionals echoed that patients were more active than usual. However, there were no significant relations between patients’ first choice of QPLs and the number of questions asked about that topic during consultations. Patients requested to see the patients’ rights video most often, and overall rated the videos as easy to understand, useful, and informative. Most of the patients reported that they like to use the tool in the future.

Aligned with the study by Walczak et al [29], migrant patients with cancer in this study evaluated the QPLs as easy to understand and quite useful. This is likely to be the result of the fact that the QPLs served the function of breaking down all cancer-related information that existed in small segments. By doing so, large chunks of information were labeled, which helped migrant patients to identify their concerns and needs more easily, thus preventing the possibility of experiencing cognitive overload [29]. Nonetheless, the evaluation became less positive with an increase in patients’ age. The older the patient, the less easy they found using the tool, which might partly be explained by a decrease in one’s cognitive competence over time and the digital divide (ie, a disparity in using digital technologies between young and old migrants and nonmigrants) [30-32]. To be readily able to adopt eHealth tools, older patients first have to be in a physically good condition and, second, be cognitively competent enough to develop internet literacy [33]. On the basis of the results of a systematic review, a suggested solution to help (older) migrant patients to better understand how QPLs work would be through incorporating a training component for patients [34]. In our intervention, only health professionals were trained. However, this—and even more in-depth training—is not sufficiently useful for patients because language barriers professionals are limited in their ability to pass on their training to patients. Therefore, a training component specifically targeted for patients is needed not only to ensure that the consultation sessions would be effectively guided by the QPLs but also to offer guidance for older migrant patients who might need help using the tool owing to old age [35]. This component can perhaps be added after each consultation session and shared with family members of the patient so that older migrant patients and their relatives are exposed to the tool more frequently, which in turn helps to increase their familiarity with the tool and enable relatives to help their older family members in using it.

Results collected to examine patient participation during the consultation sessions revealed that professionals were more active than patients in terms of leading the conversation and asking questions. A recent study revealed that patients’ passive participation could be owing to the knowledge and language barriers they face [36], also indicated by the lack of relation between the choice of QPLs and the type of questions asked. Although an interpreter or a companion was present during most of the consultation sessions, it is still possible that the patients had a lack of understanding about the subject matters raised by the professionals and/or did not have the ability to ask questions about the topics they indicated to want to discuss by their QPL choice because of an unresolved language barrier during the consultations. As a result of these barriers, there is a possibility that patients possessed insufficient competence to formulate the exact question or response they would like to make, especially when it comes to disagreeing with a statement proposed by the professional, as this requires more effortful processing, compared with simply agreeing with a statement [36,37]. As such, they may experience a lack of confidence and choose to refrain from making their point, thus generating fewer questions and talk, leading to a relatively passive outcome in terms of patient participation. As indicated by Cegala and Post [38], a lack of response from the patients’ end hinders the active adoption of a more patient-centered communication from the professionals’ end. This shows that there is a need for patients to be more empowered to secure a consultation session with active patient participation [38].

Given that the results indicated that most questions raised were about treatment-related information, a possible solution to tackle patients’ passiveness would be through distributing information related to their cancer diagnosis in their native languages along with the QPLs before each consultation session, if they had a preference for this information. This approach can enhance patients’ knowledge of the subject, which, in turn, allows them to become more confident in expressing their views and opinions about the professionals’ suggestions and thus facilitate patient-centered communication, resulting in active patient participation. In addition, working with professional interpreters instead of nonprofessional interpreters during consultations is recommended, as most patient rights topics discussed during the consultations were related to patients’ need for a professional interpreter. This will lead to better translations and enhance patients’ understanding of the conversation [39,40], enabling more active participation during consultations.

The assessment of migrant patients with cancer of their QPL-incorporated consultation sessions was fairly positive, with patients expressing high satisfaction and concluding that the QPLs helped to impact their consultation sessions positively. Professionals’ overall evaluation of patients’ participation was less satisfactory, possibly because patients did ask fewer questions and contributed less to the conversation than they might have expected as a result of the intervention. On the other hand, for patients, the QPLs were able to help them identify topics of interest in a more direct manner, and, in turn, this helped professionals to formulate a clearer picture of their unmet instrumental and/or affective needs and prepare the consultation sessions based on this feedback. Although only marginally significant, the positive relationship between patients’ choice of the patient rights QPL and health care professionals’ questions about patient rights seems to lend some substantiation for this positive effect of QPLs, helping both parties to bring up the topic of patient rights more immediately during their consultation, thus increasing patient satisfaction with the consultation sessions [34]. Corresponding with previous research, this indicates that using QPLs allowed for more effective communication between patients and professionals, as indicated by the perceived higher than usual patient participation, helping to improve the consultation sessions and encourage active patient participation [34,41].

Finally, both patients and professionals evaluated educational videos positively. Patients reported fairly high levels of identification with the characters in the narrative videos and expressed moderate intentions to watch similar videos in the future. High levels of identification with the character in the videos might likely have helped migrant patients with the ease of processing information presented because of sharing a common background with the characters [42]. Ultimately, this leads to more informed and empowered patients and, in turn, results in positive attitudes toward videos and better quality in health communication and care. What was especially interesting here was that the results reflected the patients showing more interest in narrative videos that covered the theme of patient rights in comparison with the other themes. This preference was also reflected in the selection of themes in the QPLs. Corresponding to previous research, this shows that there is a possibility that migrant patients often feel that they are not taken seriously, and to a certain extent, even discriminated against by their doctors [5,43]. Nonetheless, the precise concerns migrant patients might have concerning their rights are still scarcely researched and, thus, pointing to the fact that more empirical evidence is needed to understand inadequacies in the health care system for older migrant patients. Overall, this warrants that future research is needed to determine the underlying motivations and reasons behind choosing this theme.

### Study Limitations and Future Research

Although older migrant patients with cancer showed some intentions to use the oncological module in the future, the results indicated that older patients had concerns and expressed little ease and confidence in their ability to use the tools on their own, especially regarding using the QPLs. Again, this shows that training patients remains a crucial component of such intervention to be effective, as this study did not sufficiently target the issue of adopting and adhering to the eHealth tool, which is especially important among older adults because of their low internet self-efficacy [44]. Previous research has indicated that just short-term training can lead to successful information and communication technology adoption and outcomes for ethnically diverse older people [45]. In addition, future studies could incorporate the extended TAM and senior technology acceptance model (STAM) into the developmental process [44,46] instead of using the original TAM that was used in this study. The extended TAM sheds light on several spearheads, such as the amount of text used, page organization, and the incorporation of offline support to increase the perceived ease of use of an eHealth tool among older adults [44], whereas the STAM also incorporates age-related health and ability characteristics, such as gerontechnology self-efficacy [46]. Taking these additional factors suggested by the extended TAM and STAM into consideration, this might further increase the tool’s overall user-friendliness and lead to higher levels of perceived ease of use, usefulness, and intention to use.

Furthermore, as there is a dearth of research assessing digital literacy skills among older migrant patients, future studies are needed to gain more insight into their (lack of) competence to use new technologies in everyday life [47] and what is needed to enhance their skills. The results of such studies will help future intervention developers to gain a deeper insight into understanding what features are indeed appropriate and easy to use. The combination of incorporating the extended TAM and measuring older patients’ digital literacy might help to produce higher levels of adoption and adherence to QPLs, which ultimately increases patient participation during the consultation sessions.

Another limitation of this study is related to the developmental process of the prototype. The STAR model was designed fundamentally to promote healthy behaviors among young adolescents by encouraging them to actively participate in the e-tool developmental process [48]. This involves all stakeholders being present in the discussions and decisions taken during all developmental phases. Although we did include patients in as many phases as possible, this approach was deemed unfeasible in the *do* phase when the prototype was developed, given the fact that our target group is rather vulnerable because of their sickness and old age. As the inclusion of people who represent different areas of expertise could increase the effectiveness of brainstorming sessions largely [49], future studies should explore the possibility and feasibility of inviting ethnic minority patients who are not ill yet or migrant patients’ close relatives to take part directly in this phase of the intervention development. This could help eHealth developers to obtain a fuller picture of the attributes that can be added to the module and help maximize the customization of the tool for the target user.

### Conclusions

We conclude that QPLs make the oncological module a beneficial tool to assist health care professionals in question-asking during consultations and to better fulfill the instrumental and affective needs of older migrant patients with cancer. In addition, the use of narratives to inform older and low-literate migrant patients with cancer about health-related topics in their mother tongue is a viable approach to increase the effectiveness of health care communication with this target group. However, given that older migrant patients are less able to use the QPLs on their own, health care professionals should also look into the feasibility of adding a training component to offer offline guidance in navigating the QPLs. Finally, the oncology module developed in this study is a promising tool for both patients and health care professionals.

## Appendix

Multimedia Appendix 1 An infographic depicting the difference between the existing Health Communicator tool and the new oncological module developed in this study.

## Abbreviations

GP: general practitioner
PICS: perceived involvement in care scale
QPL: question prompt list
STAM: senior technology acceptance model
STAR: Spiral Technology Action Research
TAM: technology acceptance model
