# Supplementary figures and images for: Development and Evaluation of a Digital Intervention for Fulfilling the Needs of Older Migrant Patients With Cancer: User-Centered Design Approach
Source: J Med Internet Res. 2020 Oct 26;22(10):e21238. doi: 10.2196/21238 (PMC7652697; doi:10.2196/21238)

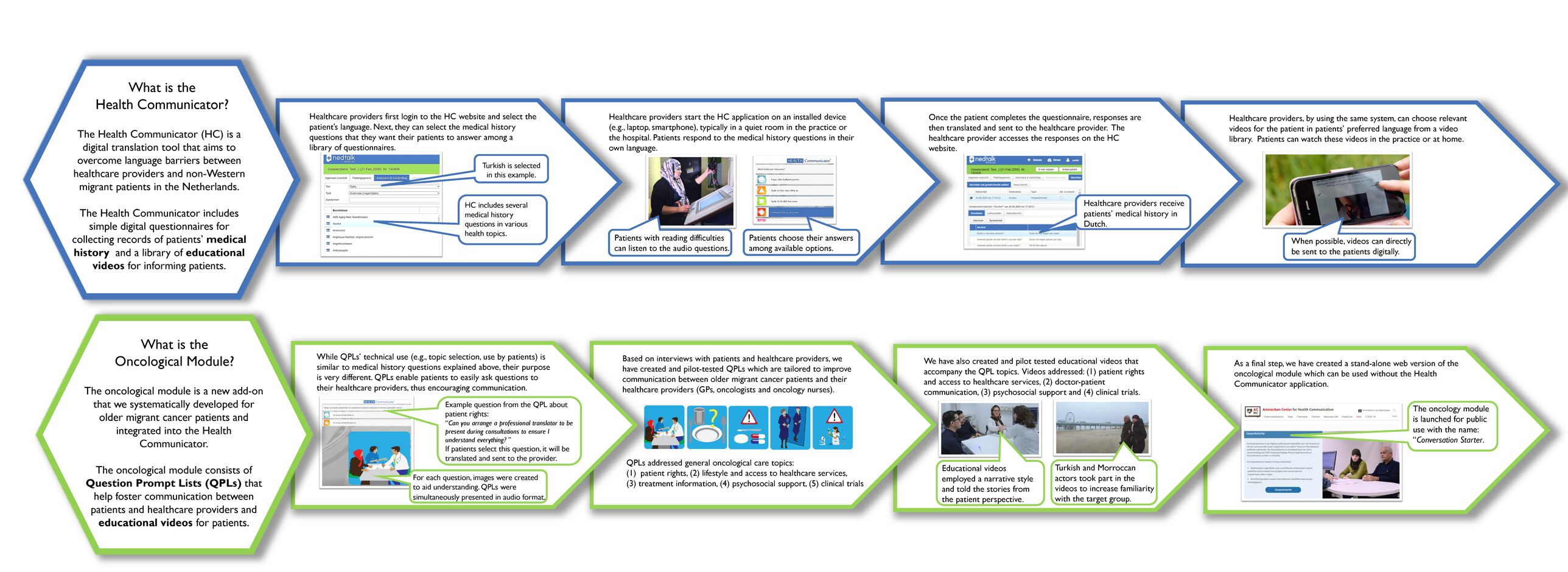

Supplement: Multimedia Appendix 1 [file jmir_v22i10e21238_app1.png]
